# American election results at the precinct level

**DOI:** 10.1038/s41597-022-01745-0

**Published:** 2022-11-03

**Authors:** Samuel Baltz, Alexander Agadjanian, Declan Chin, John Curiel, Kevin DeLuca, James Dunham, Jennifer Miranda, Connor Halloran Phillips, Annabel Uhlman, Cameron Wimpy, Marcos Zárate, Charles Stewart

**Affiliations:** 1 Massachusetts Institute of Technology, Department of Political Science, Cambridge, MA 02139 USA; 2 University of California, Berkeley, Department of Political Science, Berkeley, CA 94720 USA; 3 Ohio Northern University, Department of Political Science, Ada, OH 45810 USA; 4 Harvard University, Department of Government, Cambridge, MA 02138 USA; 5 Georgetown University, Center for Security and Emerging Technology, Washington, DC 20057 USA; 6 Arkansas State University, Department of Political Science, Jonesboro, AR 72401 USA; 7 Massachusetts Institute of Technology, Department of Electrical Engineering and Computer Science, Cambridge, MA 02139 USA

**Keywords:** Politics, Government

## Abstract

We describe the creation and quality assurance of a dataset containing nearly all available precinct-level election results from the 2016, 2018, and 2020 American elections. Precincts are the smallest level of election administration, and election results at this granularity are needed to address many important questions. However, election results are individually reported by each state with little standardization or data quality assurance. We have collected, cleaned, and standardized precinct-level election results from every available race above the very local level in almost every state across the last three national election years. Our data include nearly every candidate for president, US Congress, governor, or state legislator, and hundreds of thousands of precinct-level results for judicial races, other statewide races, and even local races and ballot initiatives. In this article we describe the process of finding this information and standardizing it. Then we aggregate the precinct-level results up to geographies that have official totals, and show that our totals never differ from the official nationwide data by more than 0.457%.

## Abstract (summary)

**Table:** 

Measurement(s)	Election results
Technology Type(s)	Scripts for collection, cleaning, and quality assurance

## Background & Summary

American election results are widely available at the largest relevant geography: governments and news outlets publish statewide vote counts for presidential, gubernatorial, and US Senate elections, while races for the US House of Representatives and for state legislatures are commonly released at the electoral district level^1,2^. In contrast, each state can choose whether and how to publish election results at lower administrative levels, so it is much more difficult to acquire, standardize, and audit the accuracy of these data. The most granular election results that states share are vote counts in each precinct, which is a geographical unit close to the level of a single polling location, typically containing a few hundred or a few thousand voters. Since 2016, we have collected and standardized the precinct-level results of national general elections, producing datasets for the 2016, 2018, and 2020 contests. Together these datasets contain over 36,000,000 rows, most of which represent the vote totals for a unique candidate-precinct combination.

Granular vote count data are required for many important questions. Local election results are widely used in quantitative political science^3–5^, and one classic application is the study of legislative districts and gerrymandering reform^6–10^. These data also have applications across many empirical sciences. Prominent applications of precinct- or county-level election results include modeling public health outcomes, particularly related to COVID-19^11–16^; local-level analyses of municipal spending, policing and crime reporting, the effectiveness of public communication, and the usage or regulation of land, water, and energy^17–23^; estimating neighbourhood-level demographics^24^; modeling small-scale labour markets or the effects of macro-economic events^25,26^; and even demonstrating how a new method in statistics or data science can be applied to important questions^27,28^.

However, it can be extremely difficult to acquire usable precinct-level election results. There are nearly 180,000 precincts across the 50 states and the District of Columbia; in every election, many candidates compete for numerous public offices in each of these precincts. In a general election there can be on the order of ten million unique combinations of precincts and candidates. But American election administration is decentralized, with election results reported individually by each state. States and localities construct precincts in very different ways, and some states change their number and composition annually without any clear means of matching them^29^. States then release election data in inconsistent formats that require customized cleaning to merge into a standardized dataset, and these results frequently contain issues that must be identified and corrected, especially for any analysis that concerns geography or spans multiple elections.

Perhaps the first effort to create national datasets of precinct-level election results was The Record of American Democracy^30^, followed by partisan efforts in the 2000s^31^. The Harvard Election Data Archive released precinct-level data for elections between 2002 and 2012, and OpenPrecincts and the Voting and Election Science Team have curated maps of precinct-level vote counts. The Redistricting Data Hub coordinated granular data collection efforts from many sources to help support public efforts to influence the post-2020 redistricting process in the United States^32,33^. Nevertheless, granular election data remain quite rare. Ours is the only effort since 2012 that has produced cleaned, standardized, quality assured, nearly complete, and freely available precinct-level election results for all states and for all offices for which the data are available. Ours is also the only precinct-level vote-count project that preserves information about the *mode* in which the votes were cast (in-person on Election Day, in-person before Election Day, and absentee), when available.

Table 1 shows the number of precincts and candidates in our datasets, broken down by the major types of electoral contest: presidential, congressional, gubernatorial, state legislative, judicial, other statewide, local, and referenda. We believe that our data include nearly every vote-getting candidate above the municipal or county level, in nearly every precinct in the country, with just three states missing: Indiana in 2018 and 2020, and New York in 2018, which did not release sufficiently complete or accurate precinct-level results (we do include states, like New Jersey and Maine, which reported township-level data). In addition to major legislative races from tens or hundreds of thousands of precincts, these data also include at least some results for regional positions like county courts and railroad commissions; ballot questions and referenda; very local races like schoolboard trustees and town aldermen; and some meta-information like the number of registered voters or absentee ballot return rates. In this article we describe the process of acquiring, standardizing, and assuring the quality of these data. We also show that when our hyper-local data are aggregated up to the national level, they are extremely close to the official results.

**Table 1:** The extent of the data about different types of races.

Race type	Precincts 2020	Candidates 2020	Precincts 2018	Candidates 2018	Precincts 2016	Candidates 2016
President	176,618	536	—	—	182,669	801
US Senate	74,181	192	132,926	385	140,657	223
US House	171,476	1,830	172,874	1,487	155,787	1,356
Governors/Lt. Governors	12,923	324	139,103	781	22,887	87
State senates	102,180	5,477	85,839	2,961	90,492	2,146
State houses	166,599	12,491	155,634	9,647	128,877	7,669
Judicial races	64,287	2,151	74,639	5,296	69,672	1,727
Other state-wide races	48,024	1,074	120,528	1,354	141,726	12,277
Local races	9,168	1,129	138,195	27,165	44,748	15,627
Ballot questions	90,034	—	85,681	—	84,553	—
Meta-information	77,953	—	75,818	—	5,618	—

The figures for President, US Senate, US House, governors, state senates, and state houses are precise counts. The other figures are not exact because they concern too many millions of rows, with thousands of unique custom descriptions of ballot questions or races at regional and local levels, but they are estimates based on a combination of string searches and manually classifying the descriptions of the race provided by each state. Large variations between years in some offices partly reflect real differences in how much information our data contains for each office across the different years, but are also partly attributable to data being reported in quite different ways in different years, with some states for example splitting the same amount of information into more precincts in one year than in another. States sometimes report ballots in aggregations that are not exactly geographic precincts, and we retain that information, so these figures are somewhat larger than the number of literal geographic precincts; for example, some counties report all of their absentee ballots as though they correspond to an “absentee ballot precinct”, and we retain that fictitious precinct alongside geographic precincts.

## Methods

### Data acquisition and completeness

In order to include a state’s election results in our datasets (from now on we will use “state” to mean “state or Washington, D.C.”), the first challenge is to find reported election results which include all of the following information: geographic data down to the precinct level (precinct, county, and state names, and standard numerical identifiers where applicable), the public office that the race is for (like the presidency or the US Senate), the candidate’s name, their political party, the mode by which the votes were cast (such as absentee or provisional ballots), the number of votes, and the stage of the election (such as whether this is a general election, a primary election, or a run-off). The 2016 data have several other types of identifiers for candidates and geographies. The variables that contain this information (36 variables in 2016, 25 variables in 2018 and 2020) are listed in Table 2.

**Table 2:** The variables in our datasets and their meanings.

Years	Name	Meaning
all	precinct	The name of the precinct
all	candidate	The name of a candidate who received votes in that precinct
all	votes	The number of votes the candidate received in the precinct
all	office	The name of the public office to which the candidate is seeking election
18/20	party_detailed	The full name of the candidate’s political party
18/20	party_simplified	Just the major parties, with others marked as “other”
2016	party	The party name, not split into detailed and simplified in the 2016 data
all	mode	The type of vote cast, like “absentee” or “provisional”
all	writein	Whether or not the votes are write-in votes
18/20	magnitude	How many candidates people can vote for in that election
all	stage	The part of the election, like whether it is a general or primary election
all	special	Whether or not the election is a special election
18/20	date	The date of the election
all	year	The election year
all	jurisdiction_name	Name of the next local government up (township or county)
18/20	jurisdiction_fips	The jurisdiction’s FIP Series (FIPS) code
all	district	The precinct’s electoral district for that office
all	county_name	The name of the county that the precinct is in
all	county_fips	The county’s FIPS code
2016	county_ansi	The county’s American National Standards Institute (ANSI) code
2016	county_lat	The county’s latitude
2016	county_long	The county’s longitude
all	state	The name of the state that the precinct is in
all	state_po	The state’s official postal code abbreviation
all	state_fips	The state’s FIPS code
18/20	state_cen	The state’s census code
all	state_ic	The state’s Inter-university Consortium for Political and Social Research code
18/20	dataverse	An indicator cataloguing where we store the data in our public repository
18/20	readme_check	An indicator that an issue with the state’s data is noted in the README file
2016	candidate_normalized	Standardized candidate name (candidate is not standardized in 2016)
2016	candidate_party	The candidate’s official party (can be distinct from nominating party)
2016	candidate_last	The candidate’s last name
2016	candidate_first	The candidate’s first name
2016	candidate_middle	The candidate’s middle name
2016	candidate_full	The candidate’s full name
2016	candidate_suffix	The suffix of the candidate’s name, if they have one
2016	candidate_nickname	The candidate’s nickname, if they have one
2016	candidate_opensecrets	The candidate’s OpenSecrets ID
2016	candidate_wikidata	The candidate’s WikiData ID
2016	candidate_fec	The candidate’s Federal Election Commission ID
2016	candidate_fec_name	The candidate’s name as listed with the Federal Election Commission
2016	candidate_google	The candidate’s Google Knowledge Graph ID
2016	candidate_govtrack	The candidate’s GovTrack ID
2016	candidate_icpsr	The candidate’s ICPSR ID
2016	candidate_maplight	The candidate’s MapLight ID

Some of the variable names are slightly different between 2016 and the other two datasets, but not enough to be ambiguous.

How do we acquire all of this information? The preferred source of election results is each individual state in the country. The reason is that administration of elections in the United States is highly decentralized, and states are responsible for counting and reporting the votes that were cast in any election, even elections for federal offices. However, states vary widely in their data reporting practices.

The simplest situation is when a state supplies a single file that can be downloaded by the public and contains enough information to fill out all of the required variables listed in Table 2. However, data collection is almost always more complicated than that. Sometimes states collect precinct-level data, but do not distribute them publicly, and they may or may not supply those data upon request. Even when they are publicly available, they might not be supplied *as datasets*; for example, results are sometimes reported only in a web app, which must be parsed using a browser to translate the results into dataset format. If data are not available online, it is sometimes possible to obtain them by directly contacting the state government, though sometimes states cannot share all of the necessary data even on request. Election results also often omit essential data, such as which office a candidate is running for, or what party a candidate is affiliated with. Frequently, election results are reported individually by sub-state bodies like county governments, and then aggregated by the state. In these cases the data for each county might use systematically different styles for recording the same information, or make independent mistakes such as typographical errors in candidate names, and an entire county may even be missing because it did not report precinct-level results to the state. Then, any essential data that are missing must be joined on from some other source. States will also often split their data into multiple files, for example by supplying all the results for the US Senate election in one file and all the results for governor in another file, and it is common to split results up by geographical divisions like counties. In many cases states will do both, and offer a separate file for every office in every county, or a separate file for every congressional district. Sometimes this means that there are dozens of files that need to be combined, which is not always a simple situation of automatically merging them, since these files may or may not share exactly the same format.

In order for us to use the data from a state, the state must supply a certain minimum amount of information. Geographically, we require a unique identifier for every precinct; from there, we can infer all of the necessary identifying information for larger geographies like counties. For every race, the state also needs to provide candidate names, and the number of votes received by those candidates. In the case of ballot questions, we require the options on the ballot, which we track as if they were candidates running for an office (so that, for example, a ballot question of the form “Should the tax on X be raised?” will have candidate name values corresponding to the options people can choose, such as “YES” and “NO”). The remaining information can be constructed using lists that are generally publicly available. For example, in some cases candidates’ parties might not be included in the precinct-level data, and in those cases they need to be merged in using other publicly available datasets.

Because of the number and severity of possible data problems, in some cases a state simply does not provide sufficiently complete or accurate precinct-level data. In such cases, raw precinct-level election results are sometimes available from OpenElections, an organization that coordinates the collection of election results using a combination of automatic scraping, contacting state and local governments, and entering data manually^34^ (the OpenElections effort is distinct from ours in that they focus on collecting precinct-level election results, often acquiring data in cases where intensive work is required to obtain the data from states or even from county-level governments, whereas we focus on standardizing and quality assuring the data).

The sources of our data, and the formats that we acquired the raw data in, are described in Table 3 for the two more recent elections in our dataset (2020 and 2018, which were a presidential and a midterm election year respectively). The table summarizes where the original datasets were acquired, whether the election results themselves omitted any of the necessary information and needed to be combined with other data, what format the data were supplied in, and whether the datasets were separated out by some characteristic below the state level and had to be merged to create a statewide dataset.

**Table 3:** Data sources.

State	Source 2020	Availability 2020	Added 2020	Type 2020	Split 2020	Source 2018	Availability 2018	Added 2018	Type 2018	Split 2018
AK	gov	download	geo	csv		gov	download		csv	
AL	gov	download		xls	geo	gov	download		xls	geo
AR	gov	download		xls	geo, office	gov	download		xls	geo, office
AZ	gov	download		xml		gov	download		tab	geo
CA	gov	download		csv		OE	download	geo	csv	geo
CO	gov	download		xls		gov	download	cand	xls	
CT	gov	download		csv	geo, office	gov	download		csv	geo, office
DC	gov	download		multi		gov	download		multi	
DE	gov	web only	geo		office	gov	download	cand	csv	
FL	gov	download		pdf	geo	gov	download		tab	
GA	gov	download		csv		gov	download	geo	csv	stage
HI	gov	download		csv		gov	download		csv	
IA	gov	download	geo	xls	geo, office	gov	download		xls	geo, office
ID	gov	download		xls	office	gov	download		xls	
IL	gov	download		csv	geo	gov	download	geo	csv	geo
KS	gov	request		xls		gov	download	geo	xls	
KY	gov	download		xls	geo	gov	web only			
LA	gov	download		xls		gov	download		xls	geo, office
MA	gov	download		csv	office	gov	download		csv	office
MD	gov	download		csv		gov	download		csv	geo
ME	gov	download		xls		gov	download		xls	office
MI	OE	download		csv		gov	request		tab	multi
MN	gov	download		xls		OE	download		csv	
MO	OE	download		csv		OE	download		csv	
MS	OE	download		csv		OE	download		csv	
MT	gov	download		xls	geo	gov	download		xls	
NC	gov	download		csv		gov	download		tab	
ND	gov	web only			office	gov	download		multi	geo, office
NE	gov	web only	office		office	gov	download		xls	geo, office
NH	gov	download	geo	xls	geo, office	gov	download	geo	xls	geo, office
NJ	gov	download	geo	pdf	geo	OE	download		csv	geo
NM	gov	download	geo	xls	office	gov	download		xls	office
NV	gov	download	party	multi		gov	download	party	multi	
NY	OE	download		csv		OE	download		csv	
OH	gov	download		xls		gov	download		xls	office
OK	gov	download		multi		gov	download		multi	
OR	OE	download		csv		OE	download		csv	
PA	gov	request		csv		gov	request		csv	
RI	gov	web only			geo, office	gov	web only			geo, office
SC	gov	download		csv		OE	download		csv	
SD	gov	download		pdf		OE	download		csv	
TN	gov	download		xls		gov	download		xls	
TX	gov	download		csv	office	gov	web only	multi		office
UT	OE	download		csv		OE	download		csv	geo
VA	gov	download		csv		gov	download		csv	
VT	gov	download		xls	office	OE	download		csv	
WA	gov	download		csv		gov	download		csv	geo
WI	gov	download		xls	office	gov	download		xls	office
WV	OE	download		csv		OE	download		csv	
WY	gov	download		pdf	geo	gov	download		pdf	geo

Source: gov = government, OE = OpenElections. Avail.: download = public, request = by request, web = in-browser. Added: missing geographic, office, party, or candidate details. Type: multi = multiple formats. Split: how files are split up.

Table 3 shows that, in 2020, we were able in 42 out of 51 cases to acquire high quality precinct-level election data from the branch of the state government that is primarily associated with administering elections (and in California, those data were available from the state’s redistricting commission). In 7 of the remaining 8 cases, we acquired data from OpenElections. There is just one case, Indiana, for which we could not recover precinct-level election results in 2020 (many of Indiana’s counties simply did not report precinct-level election results in 2020). In 2018, 38 datasets were available from state election administrations. Of the remaining 13 datasets, we have published 11 using data from OpenElections. The remaining two are Indiana and New York, for which the available data have sufficiently incomplete or inaccurate vote counts that they are still pending final cleaning and quality assurance. In these counts we have included states like New Jersey and Maine, where data were released at the township level, as nevertheless successfully releasing sufficiently granular elections data to be included in our dataset.

In addition to listing where the data were acquired, Table 3 also identifies any supplementary sources needed to fill in missing information, and what format the data were originally in. The table shows that there is a wide range in how ready the original datasets are for cleaning, standardizing, and incorporating into a national dataset. Hawaii is an example of a particularly straightforward state: in both 2018 and 2020, the state government publicly posted a single comma-separated file that contained election results for every candidate in every precinct. But in several states we found the opposite extreme, in which data were made available only through in-browser web apps or PDFs, split up by office and county, and missing crucial information.

Some of the information in Table 3 mirrors Tables A1 and A2 in a description of OpenElections data sources^34^, but Table 3 includes information not in those tables and *vice versa* (and some differences between the two tables showcase the fluidity of states’ reporting practices, which might change even across a few weeks within the same election).

### Standardization

Once a state’s data have been collected and merged into a single spreadsheet, the next task is to standardize them. In order for the datasets to be merged into one national dataset, they need to contain the same variables, and many variable values also need to be standardized.

To see why variable values may need to be standardized, consider for example the case of candidate names. Different states, and even the different reporting units within one state, may submit very different formatting of candidate names. The reason is that candidate names are often entered manually by different people in different reporting areas, and in addition to typographical errors, the person responsible for entering these data in one area might choose a different convention than someone in another area.

In 2020, for example, Pennsylvania recorded a vote for President Biden in its presidential contest as a vote for the candidate named “BIDEN, JOSEPH ROBINETTE”, whereas Arksansas recorded it as a vote for “Joseph R. Biden/Kamala Harris”. These names need to match across states. But the same problem might occur at the county level, or it might occur in just the cases where a person who entered the data made one systematic mistake. In Georgia’s 2020 precinct data, for example, the name of US Senate candidate Matt Lieberman appeared in 14,035 rows, but in Franklin County his name was misspelled 40 times as “Matt Lierberman”. That raw data file contained 774,906 rows, so a mistake that occurs in one candidate name 40 times cannot be reliably identified by eye. The number of candidates listed in Table 1, and the number of precincts those candidates appear in, demonstrates the scale of this challenge: candidate name matching is not a matter of simple find and replace actions, but requires automated fuzzy matching across the whole dataset. So, we first convert all candidate names to uppercase (a practice that we adopted in our 2018 and 2020 data), which greatly reduces the number of errors in matching, without meaningfully reducing the usability of the dataset. Then we use the default extract function from the process module of the fuzzywuzzy Python library, with a sensitivity cutoff that typically shows every collection of strings which either contain large amounts of shared text, or are separated by a few substitutions or insertions.

In addition to standardizing variable values, we also standardize what variables are included. Often the variables of interest need to be inferred, or are combined into one variable and must be teased apart. For example, a state may provide the precinct designation as “countyName_precinctName”, and then the precinct name and county name need to be split into two different variables, and the standard county FIP Series (FIPS) code added. Another common issue is that party names, or the word “writein”, are often included in the raw data as part of a candidate name, but we store each of these in a separate variable.

Finally, in some rare cases, states or other research organizations release shapefiles that map the boundaries of the precincts in a state. These spatial data are particularly important for studying the spatial problem of redistricting. When such files are available, we attempt to retain any geographic information, such as the particular formatting of precinct names, that would be needed to match our precinct-level election results onto the precinct shapefiles. When such data are not available, we avoid removing information from precinct names whenever possible, in the interest of making it possible to match to any geographic files that might be made available after we release our data; excising information that might initially appear unnecessary might hinder attempts by end-users interested in matching our tabular data to spatial precinct data that subsequently becomes available.

Importantly, our standardization efforts are aimed at standardizing data *within* election years, but there is no general way to match precinct-level election results *across* election years. There is no requirement for a precinct to have the same name from one election to the next, and states may create, abolish, or rename precincts between elections, with or without providing a crosswalk file that matches new precinct names to old precinct names. Worse, precinct boundaries frequently change, so a precinct with the same name in roughly the same location may contain a different population from one election to the next^32^. We therefore emphasize that users of our datasets are not encouraged to join precincts by name across elections, unless they have verified with a separate source (such as the precinct shapefiles maintained by the United States Elections Project) that the name identifies the same precinct in both years and that the geography of the precinct did not change.

## Data Records

Our 2016, 2018, and 2020 precinct-level American election result datasets are each available for download on a public repository. In 2016 and 2020, the data have been published as Harvard Dataverse records. The 2018 data are available on a public GitHub account while the formatting of those datasets is being updated to the standardized format used for the 2020 data. For ease of use, each of these records are divided into datasets in several different ways.

The data records corresponding to elections in 2020 are available on Harvard Dataverse and are organized as follows: 2020 US President election results^35^, 2020 US Senate election results^36^, 2020 US House election results^37^, 2020 state-level election results^38^, and individual datasets for each state listing all of the election results there^39^.

For 2018 elections, the data are currently stored on a GitHub repository, although they will eventually be moved to the Harvard Dataverse^40^.

For 2016, the data are available on Harvard Dataverse, in the following individual data records: 2016 US President election results^41^, 2016 US Senate election results^42^, 2016 US House election results^43^, 2016 state-level election results^44^, and 2016 local-level elections^45^.

In addition to these flat files, the data are also available for download in SQL database format^46^.

## Technical Validation

We have developed a procedure for ensuring the quality of our datasets that emphasizes reproducibility, and involves many large-scale automated validation checks. In our cleaning process, one person cleans and standardizes a dataset, and then saves a copy of the code that they used. Then a second person runs a standard battery of quality assurance checks on the output of that cleaning code, and reports to the first person any issues that the quality assurance checks identified. These two people then iterate: the first person updates their cleaning code, and the other runs the quality assurance check on its output, until the checks do not find any remaining problems. Not only is the code (and every draft of the code before the finished version) saved in a GitHub repository, but the discussion of any issues that arose during the quality assurance process are also saved as comments in that repository, so that any future issues can be traced to their origin and addressed without re-doing any of the initial cleaning. Because this procedure was developed and implemented over the course of our data collection efforts, we can consistently supply replicable code and raw datasets for the 2018 and 2020 datasets, but we cannot guarantee that full replication scripts are available for the 2016 dataset.

The quality assurance process is largely implemented as an automatic engine in Python. For every variable, the engine checks that the variable exists and is formatted according to the codebook, and also identifies a variety of common data problems. Table 4 lists the checks that are performed, the purpose of each check, and the variables that each check applies to.

**Table 4:** The automated checks performed by our quality assurance engine, the purpose of each check, and the variables that they apply to.

Type	Purpose	Variables
existence	checks that all variables exist	all
character	checks for suspicious string characters	candidate, county_name, dataverse, district, jurisdiction_fips, jurisdiction_name, magnitude, mode, office, party_detailed, party_simplified, precinct, special, stage, state, state_cen, state_fips, state_ic state_po, votes, writein
similarity	checks for suspiciously similar values	county_name, county_FIPS, dataverse, district, jurisdiction_fips, juristiction_name, magnitude, mode, party_detailed, party_simplified, special, stage, state state_cen, state_fips, state_ic, state_po, writein
dictionary	checks values match a definitive list	county_fips, dataverse, date, party_simplified state_cen, state_fips, state_ic, state_po
uniqueness	checks values are unique	magnitude, office, state, state_cen, state_fips, state_ic, state_po, writein
numerical	checks numerical constraints	votes
duplicates	finds duplicated and near-duplicated rows	all

In the interest of space we list the variable names here; the meaning of these variables can be understood by consulting Table 2.

The two most important types of checks are character checks and similarity checks. Character checks identify any unexpected symbols inside a variable value; for example, it is likely a mistake if a candidate name contains a “%” symbol, or if a vote total contains a non-numeric character. There are two types of similarity checks. General similarity checks compare the values of variables to a list of common words that are frequently misspelled. For example, a race in Texas in 2020 was described as electing a “JUSTIVE” rather than a “JUSTICE”. Substitution errors in common words can be caught by comparing the strings in the raw data to a list of words that frequently appear in election data. Specific similarity checks, on the other hand, compare the values within a variable to one another. We implement these in two ways: through fuzzy string matching, and also by simply printing out the unique values of a variable in alphabetical order and noting any nearly duplicated names. In some cases this catches typographical errors, like the spelling of Matt Lieberman as “Matt Lierberman”. Many of the cases these similarity checks flag, however, are different ways of writing the same information, which need to be standardized so that the values match; for example, these checks would reveal that the name of a US Senate candidate from West Virginia is rendered variously as “Joe Manchin”, “Joseph Manchin”, and “Joseph Manchin III”.

In some cases, we have the opportunity to check variables against a dictionary of definitive values. An example is state FIPS codes, which are the numerical identifiers that the U.S. Census Bureau has assigned to each state and county. Since each state and county has just one unique correct FIPS code, we can enforce that every value of this variable is exactly correct. It is also sometimes useful to check for unique correspondences. For example, usually each type of office corresponds to just one magnitude (the number of election winners). It would be difficult to enforce that each office is associated with exactly the correct magnitude — state house races in Michigan should typically have a magnitude of 1, but state house races in New Jersey should typically have a magnitude of 2, except perhaps in the case of certain types of special elections — but a uniqueness check can identify cases where the same office within the same state corresponds to more than one magnitude value, which is likely a mistake. For numerical values, we can also check certain constraints; for example, only some numbers represent legitimate vote totals. Finally, in a dataset where each row is intended to represent a unique precinct-candidate combination, not only should no rows ever be exact duplicates of each other, but there should almost never be two rows with the same candidate name and the same precinct name (with a few exceptions, for example if a state separately reports the vote totals that an electoral fusion candidate received under each of the parties that nominated them). A final check identifies any rows that are either duplicates or near-duplicates of each other.

In addition to all of these checks, there is another crucial type of check that can be only semi-automated: aggregation checks. States release vote count totals at various higher levels of aggregation than the precinct, and the results of all races at their highest level of aggregation (the full electoral constituency) is common knowledge once election results have been certified. So, when we add together the vote totals in each precinct within a given county or state, the resulting sum should match the official vote total in that area. Of course, this does not catch every mistake, because errors might cancel each other out (over-counting a candidate’s vote total in one precinct and then under-counting that candidate’s total by the same amount in another precinct within the same county will still produce the correct county-level total), but this check will catch net errors in a candidate’s vote total across some larger geography.

Aggregating our vote counts to compare them to published results is not completely straightforward, because the format in which automated counts are published varies widely, so we have built routines for automatically aggregating vote counts to any number of a variety of common state-published formats. But there is a more serious reason that this check cannot be automated in general: the names of counties and (especially) candidates in the available county-level election results do not always exactly match their names in our final precinct-level datasets, and ensuring that such a large number of strings do match would be nearly tantamount to cleaning county-level election results in addition to the precinct-level election results.

Nevertheless, we base a simple automated first pass on the fact that it is much easier to ensure that all county names match between the files than to make all of the candidate names match. We begin by running an aggregation checking script in R that simply compares the list of vote totals that each candidate received in the official county-level data to the list of candidate-by-candidate sums over precinct-level vote totals. Without consulting *which* candidate received each vote total, it simply reports any discrepancies between those two lists. So if (for example) our precinct-level data report that there is some candidate who received 800 votes across all of the precincts in a county, and the county-level results report that there is a candidate who received 801 votes, then we can check by eye whether the datasets are referring to the same candidate, since in this example it is likely that our precinct-level vote total for some candidate differs from their county-level total by one vote. This automated first pass helps to identify counties with major problems, but of course it is not sufficient on its own to identify every issue. We therefore also compare our data to official county-level results by eye. When we find major discrepancies, we reach out to the state that provided the data (where applicable) and request clarification, and in some cases they are able to explain or resolve the discrepancy.

Counties are just one possible unit of aggregation, and indeed we can use the aggregation check idea to determine the accuracy of the precinct-level data across any larger geography. We will conclude this article by conducting aggregation checks on all races for federal office in the 2016, 2018, and 2020 datasets. However, we should be clear that there are many reasons that vote totals may be unavoidably different at different levels of aggregation. Probably the biggest reason is that states often do not officially certify precinct-level results (although when they do, we use the certified totals), and in many cases the official vote counts either include some ballots that were not included in the unofficial counts, or exclude some ballots that were included unofficially. This is especially difficult to resolve when there is legitimate uncertainty about precise vote totals; for example, votes that are the topic of pending court challenges may be legitimately counted when the precinct-level dataset is produced and equally legitimately not counted in a county-level dataset. Second, there are sometimes small clerical errors in the announced vote counts, but it is not possible to ascertain whether the precinct-level values are erroneous or the higher-level values are. Third, it is possible for the values to not match for reasons to do with how votes are reported. Some states, such as Florida, require the suppression of precinct-level results when the number of voters is below a certain threshold. Still others induce random noise into precinct returns in low-turnout precincts. Fourth, precinct-level datasets sometimes do not identify every candidate who receives a write-in vote, but those votes may be counted towards the candidate’s official vote total. Finally, even though precincts typically partition a single county that completely contains them, there are some exceptions. For these reasons and many others, it is not generally possible to create a precinct-level dataset that precisely adds up to the official county- or state-level data. However, the result certainly *should* be close. In cases where discrepancies simply cannot be avoided, we provide a readme file that identifies the discrepancy and, where possible, notes its cause and why it has not been resolved.

Despite these unavoidable problems, Table 5 shows that our precinct-level results for federal races aggregate all the way to the national level with extremely high accuracy. We check the official nationwide vote totals for both major parties in the three types of federal election — presidency, US Senate, and the US House of Representatives — against the totals obtained when summing our precinct-level data up to the national level. This generates sixteen comparisons: four presidential totals, and six each of the Senate and the House. The “accuracy” percentage *a* that we report is

$$a\equiv 100 \% -\frac{| r-f| }{f}\cdot 100 \% $$

where *r* is the sum of our precinct-level votes for relevant offices, and *f* is the official total. Table 5 shows that every one of the sixteen totals is at least 99.5% accurate, and about half are at least 99.9% accurate. Out of tens of millions of votes cast for each party in each of these contests, the discrepancies are tens of thousands of votes in most cases.

**Table 5:** The sum of our precinct-level data compared to the official nationwide vote totals for each major party in each type of federal race in 2016, 2018, and 2020.

Year	Race type	Candidate/Party	States	Our total	Official total	Percent accuracy
2016	US President	Hillary Clinton	51/51	65,851,676	65,853,514	99.997%
2016	US President	Donald Trump	51/51	62,980,405	62,984,828	99.993%
2016	US Senate	Democrats	35/35	50,680,324	50,610,704	99.862%
2016	US Senate	Republicans	35/35	40,848,585	40,788,131	99.852%
2016	US House	Democrats	51/51	59,303,971	59,327,502	99.960%
2016	US House	Republicans	51/51	60,002,678	59,945,740	99.905%
2018	US Senate	Democrats	32/34	47,576,064	47,394,573	99.619%
2018	US Senate	Republicans	32/34	31,883,643	31,895,337	99.963%
2018	US House	Democrats	49/51	55,902,331	55,648,030	99.543%
2018	US House	Republicans	49/51	47,719,862	47,589,647	99.726%
2020	US President	Joe Biden	50/51	80,035,360	80,026,508	99.989%
2020	US President	Donald Trump	50/51	72,495,664	72,486,635	99.988%
2020	US Senate	Democrats	35/35	37,706,264	37,765,395	99.843%
2020	US Senate	Republicans	35/35	39,300,424	39,257,329	99.890%
2020	US House	Democrats	50/51	76,175,504	76,159,116	99.978%
2020	US House	Republicans	50/51	70,807,544	70,727,831	99.887%

The percentage is calculated using the equation specified in the text. Here it is rounded to five digits (the smallest number of digits for which no total rounds up to 100%). The states column shows the number of states (plus DC) included in our dataset out of the number of states that the race took place in. As explained in the text, some of these states are excluded from the total in the table because partisan comparisons are not coherent in every race in the country (for example in the case of run-offs that are not general elections with simple partisan labels).

We once again emphasize that aggregation checks cannot guarantee that our data are almost completely devoid of errors, because as we have discussed precinct-level errors might cancel each other out. What this analysis does demonstrate, however, is that our data are almost completely devoid of *systematic* errors by party within major federal races.

Another caveat is that we have had to exclude some races from these comparisons. Which contests are missing and why? Indiana did not consistently release precinct-level vote totals in 2020 (some counties in Indiana did release precinct-level results, but many did not), so it is not possible to include. Our 2018 data do not currently include Indiana, New York, or the Maine House of Representatives election that was decided using Instant Runoff Voting; these are all excluded from the totals in Table 5. The totals in the table also exclude some congressional races (the 2020 races in Georgia, the 2018 pre-runoff races in Louisiana, and races in Pennsylvania) which have complicated election structures that make party-by-party comparisons to official totals infeasible, though our candidate-level totals for these races are all individually accurate. To also give a sense for the state-level variation in accuracies, Table 6 compares the sum of our precinct-level vote totals in each state to the official state-level vote total, for both major candidates in the most recent presidential election.

**Table 6:** Accuracy of our vote totals for major party presidential candidates at the state level.

State	Trump 2020 Ours	Trump 2020 Official	Trump 2020 Accuracy	Biden 2020 Ours	Biden 2020 Official	Biden 2020 Accuracy
AK	189,951	189,951	100%	153,778	153,778	100%
AL	1,441,170	1,441,170	100%	849,624	849,624	100%
AR	760,647	760,647	100%	423,932	423,932	100%
AZ	1,661,686	1,661,686	100%	1,672,143	1,672,143	100%
CA	6,006,518	6,006,429	99.999%	11,110,639	11,110,250	99.996%
CO	1,364,607	1,364,607	100%	1,804,352	1,804,352	100%
CT	714,697	714,717	99.997%	1,080,680	1,080,831	99.986%
DC	18,586	18,586	100%	317,323	317,323	100%
DE	200,603	200,603	100%	296,268	296,268	100%
FL	5,668,716	5,668,731	100%	5,297,036	5,297,045	100%
GA	2,461,837	2,461,854	99.999%	2,474,507	2,473,633	99.965%
HI	196,864	196,864	100%	366,130	366,130	100%
IA	897,672	897,672	100%	759,061	759,061	100%
ID	554,119	554,119	100%	287,021	287,021	100%
IL	2,446,891	2,446,891	100%	3,471,915	3,471,915	100%
KS	771,406	771,406	100%	570,323	570,323	100%
KY	1,326,418	1,326,646	99.983%	772,285	772,474	99.976%
LA	1,255,776	1,255,776	100%	856,034	856,034	100%
MA	1,167,202	1,167,202	100%	2,382,202	2,382,202	100%
MD	976,414	976,414	100%	1,985,023	1,985,023	100%
ME	360,737	360,737	100%	435,072	435,072	100%
MI	2,649,234	2,649,852	99.977%	2,801,469	2,804,040	99.908%
MN	1,484,065	1,484,065	100%	1,717,077	1,717,077	100%
MO	1,718,736	1,718,736	100%	1,253,014	1,253,014	100%
MS	756,764	756,764	100%	539,398	539,398	100%
MT	343,602	343,602	100%	244,786	244,786	100%
NC	2,758,773	2,758,775	100%	2,684,292	2,684,292	100%
ND	235,595	235,595	100%	114,902	114,902	100%
NE	556,846	556,846	100%	374,583	374,583	100%
NH	365,660	365,660	100%	424,937	424,937	100%
NJ	1,883,313	1,883,274	99.998%	2,608,400	2,608,335	99.998%
NM	401,860	401,894	99.992%	501,552	501,614	99.988%
NV	669,480	669,890	99.939%	703,186	703,486	99.957%
NY	3,252,233	3,244,798	99.771%	5,245,067	5,230,985	99.731%
OH	3,154,834	3,154,834	100%	2,679,165	2,679,165	100%
OK	1,020,280	1,020,280	100%	503,890	503,890	100%
OR	958,448	958,448	100%	1,340,383	1,340,383	100%
PA	3,379,321	3,377,674	99.951%	3,457,343	3,458,229	99.974%
RI	199,922	199,922	100%	307,486	307,486	100%
SC	1,385,103	1,385,103	100%	1,091,541	1,091,541	100%
SD	261,043	261,043	100%	150,471	150,471	100%
TN	1,852,475	1,852,475	100%	1,143,711	1,143,711	100%
TX	5,889,022	5,890,347	99.978%	5,257,519	5,259,126	99.969%
UT	865,139	865,140	100%	560,282	560,282	100%
VA	1,962,430	1,962,430	100%	2,413,568	2,413,568	100%
VT	112,797	112,704	99.917%	242,828	242,820	99.997%
WA	1,584,651	1,584,651	100%	2,369,612	2,369,612	100%
WI	1,610,065	1,610,184	99.993%	1,630,673	1,630,866	99.988%
WV	548,463	545,382	99.435%	235,984	235,984	100%
WY	193,559	193,559	100%	73,491	73,491	100%

Ours is our total, official is the official total, and accuracy is the accuracy percentage.

## Usage Notes

Readmes are provided alongside the datasets and specify three important types of information. First, they describe any errors or data limitations that were identified in the process of collecting, standardizing, and assuring the quality of the data, but which for any reason could not be resolved. An example is cases where precinct-level vote counts differed from county-level vote counts and could not be reconciled.

Second, they identify missing information. For example, we typically include vote counts for write-in candidates, but in the file for our 2020 precinct-level election results we identify some states where write-ins were not consistently reported.

Third, the readme files note the inclusion of certain types of meta-information that prevent the dataset from being immediately interpreted as containing candidate-precinct vote totals. For example, the readme for Michigan in 2018 notes that the state’s original dataset included “statistical adjustment” rows, which do not correspond to any real precinct, and have a precinct value of “9999”. Another common example is when states report data in such a way that candidates are assigned 0 votes in precincts that they did not contest, which cannot be cleaned in general, because there is often no way to differentiate at scale between those (many) precincts where a candidate legitimately received 0 votes, and precincts that a candidate did not actually contest and was nevertheless assigned 0 votes in by the state’s precinct-level data. In these cases the readme warns users to be wary of these fictitious candidate-precinct combinations when computing statistics.

## Data Availability

A large code-base was required to translate the original raw data into organized and structured datasets. This suite of state-specific files, written variously in Python, R, and Stata, is stored and retained internally. The scripts that we used to clean the data from each state in 2018 and 2020 are all available on a public GitHub repository^47^.
